# Recombinant cytokines produced in *Komagataella phaffii*: advances in design, production, and purification strategies

**DOI:** 10.1007/s11033-026-11829-4

**Published:** 2026-05-11

**Authors:** Ana C. K. Pedra, Natasha R. de Oliveira, Mara A. C. Maia, Andriele B. Madruga, Beatriz C. M. Santos, Odir A. Dellagostin, Thaís L. O. Bohn

**Affiliations:** Laboratório de Vacinologia, Centro de Desenvolvimento Tecnológico, Núcleo de Biotecnologia, Universidade Federal de Pelotas, Pelotas, RS Brazil

**Keywords:** Bioprocess, Protein production, *Komagataella phaffii*, Immunocytokines, Immunity

## Abstract

Cytokines are small glycosylated polypeptides that orchestrate immune responses and are widely produced in recombinant form for therapeutic and research purposes. This review highlights *Komagataella phaffii* as an efficient host for the heterologous expression of recombinant cytokines from human and other species. A systematic search of PubMed, Scopus, and Web of Science identified studies addressing upstream and downstream processes involved in cytokine expression. Molecular design strategies such as codon optimization, vector design, and signal peptide selection are discussed together with process parameters including temperature, inducer concentration, and bioreactor conditions, as well as recovery and purification of biologically active cytokines. Optimal production is often associated using multi-copy vectors driven by the AOX1 promoter, α-factor secretion signals, methanol induction (0.5–1.5% v/v), temperatures below 30 °C, and co-feeding strategies. Downstream purification commonly yields products exceeding 95% purity. Strategies such as PEGylation, albumin fusion, and antibody fusion are also described to improve cytokine stability and half-life. This review integrates and critically analyzes molecular and bioprocessing advances that establish *K. phaffii* as a powerful platform for recombinant cytokine production.

## Introduction

Cytokines are pivotal regulatory molecules that maintain immune homeostasis and coordinate a wide range of biological processes, including cell differentiation, proliferation, and inflammatory responses [1]. They are secreted not only by immune cells—such as monocytes, macrophages, T and B lymphocytes, and natural killer (NK) cells—but also by non-immune cells, including endothelial, epithelial, and fibroblast cells [2, 3]. Acting through specific membrane receptors, cytokines mediate highly sensitive signaling cascades that regulate gene expression and cellular behavior [3].

Given their central role in immune modulation, recombinant cytokines have been extensively investigated as therapeutic agents and as molecular tools for studying the pathophysiology of infectious, autoimmune, and neoplastic diseases [1]. Several have already been commercialized by the biopharmaceutical industry, including interleukins for renal cell carcinoma, interferon-α for hepatitis C, interferon-β for multiple sclerosis, and various colony-stimulating factors for neutropenia and bone marrow recovery [4]. However, the widespread use of recombinant cytokines in both medicine and research depends heavily on cost-effective and scalable production systems capable of maintaining biological activity and correct protein folding [5].

Prokaryotic, yeast, and mammalian cells represent the main expression platforms for recombinant proteins [6]. *Escherichia coli* remains the most widely used host due to its rapid growth, low cultivation cost, and well-characterized genetics, physiology, and metabolism [7]. Nonetheless, bacterial systems are limited by the lack of post-translational modifications, improper folding, and inclusion body formation [7]. Mammalian cells, in contrast, perform authentic post-translational modifications but are expensive to cultivate and require complex bioprocessing conditions [8]. These limitations have positioned yeast expression systems as an intermediate platform that combines cost efficiency with the ability to perform eukaryotic processing [6].

*Komagataella phaffii*, formerly known as *Pichia pastoris*, is a methylotrophic yeast extensively used as a microbial factory for recombinant protein expression [9]. As a eukaryotic host, *K. phaffii* supports correct protein folding, disulfide bond formation, and both N- and O-linked glycosylation, which are essential for producing functional therapeutic proteins such as cytokines [5]. This yeast offers key advantages: rapid growth to high cell densities, the ability to secrete target proteins into the culture medium, and straightforward scale-up to industrial bioreactors [9]. Its well-established genetic tools, including strong and regulatable promoters, have consolidated *K. phaffii* as one of the most attractive hosts for producing biopharmaceuticals [5].

The efficiency of recombinant protein expression in *K. phaffii* results from a complex interplay between genetic, physiological, and process parameters [10]. In upstream processing, factors such as codon optimization, gene copy number, promoter strength, secretion signal selection, and host strain physiology critically influence productivity. Similarly, environmental conditions, such as temperature, pH, inducer concentration, and dissolved oxygen must be carefully optimized to achieve high yields of correctly folded and active cytokines [10, 11]. Downstream processing then focuses on efficient recovery and purification strategies to ensure high purity and yield [12]. The following sections summarize and critically discuss the main molecular and bioprocessing approaches reported in the literature for the expression of recombinant cytokines in *K. phaffii.*

## Methodology

A systematic literature review was conducted to identify key strategies for recombinant cytokine production using the *K. phaffii* expression system. Searches were performed for terms appearing in article titles and/or abstracts, restricted to studies published from 2015 onward. The initial query combined “*Pichia pastoris* OR *Komagataella phaffii*” AND “recombinant cytokines”. Subsequent searches targeted specific cytokine classes, employing the following combinations: “*Pichia pastoris* OR *Komagataella phaffii*” AND “recombinant interferon”; “recombinant interleukin”; “transforming growth factor”; “hematopoietic growth factor”; and “recombinant tumor necrosis factor”.

The search was carried out in March 2025 across PubMed, Scopus, and Web of Science databases. Retrieved articles were manually screened, and those meeting the inclusion criteria were retained: (a) availability of an English abstract; and (b) reporting of expression or optimization strategies for cytokine production. The selected studies were analyzed according to the following parameters: (I) Cytokine type; (II) Target species; (III) Application; (IV) *K. phaffii* strain; (V) Promoter; (VI) Codon optimization; (VII) Gene dosage; (VIII) Signal peptide; (IX) Expression induction parameters; (X) Recovery method; (XI) Purification strategy; (XII) Yield and purity; and (XIII) Biological activity.

The information extracted from the reviewed articles was organized into a table that summarizes the upstream and downstream strategies employed, allowing a comparative assessment of the most effective combinations of genetic and bioprocessing parameters.

### Upstream processing in *Komagataella phaffii*

*K. phaffii* has emerged as a robust and versatile platform for the production of recombinant proteins at both laboratory and industrial scales [5]. This yeast combines the advantages of prokaryotic and eukaryotic systems, offering high productivity at low cost while allowing essential post-translational modifications, such as disulfide bond formation and glycosylation [11]. In addition, its capacity to secrete recombinant proteins simplifies purification and enhances process scalability [11].

**Fig. 1:**
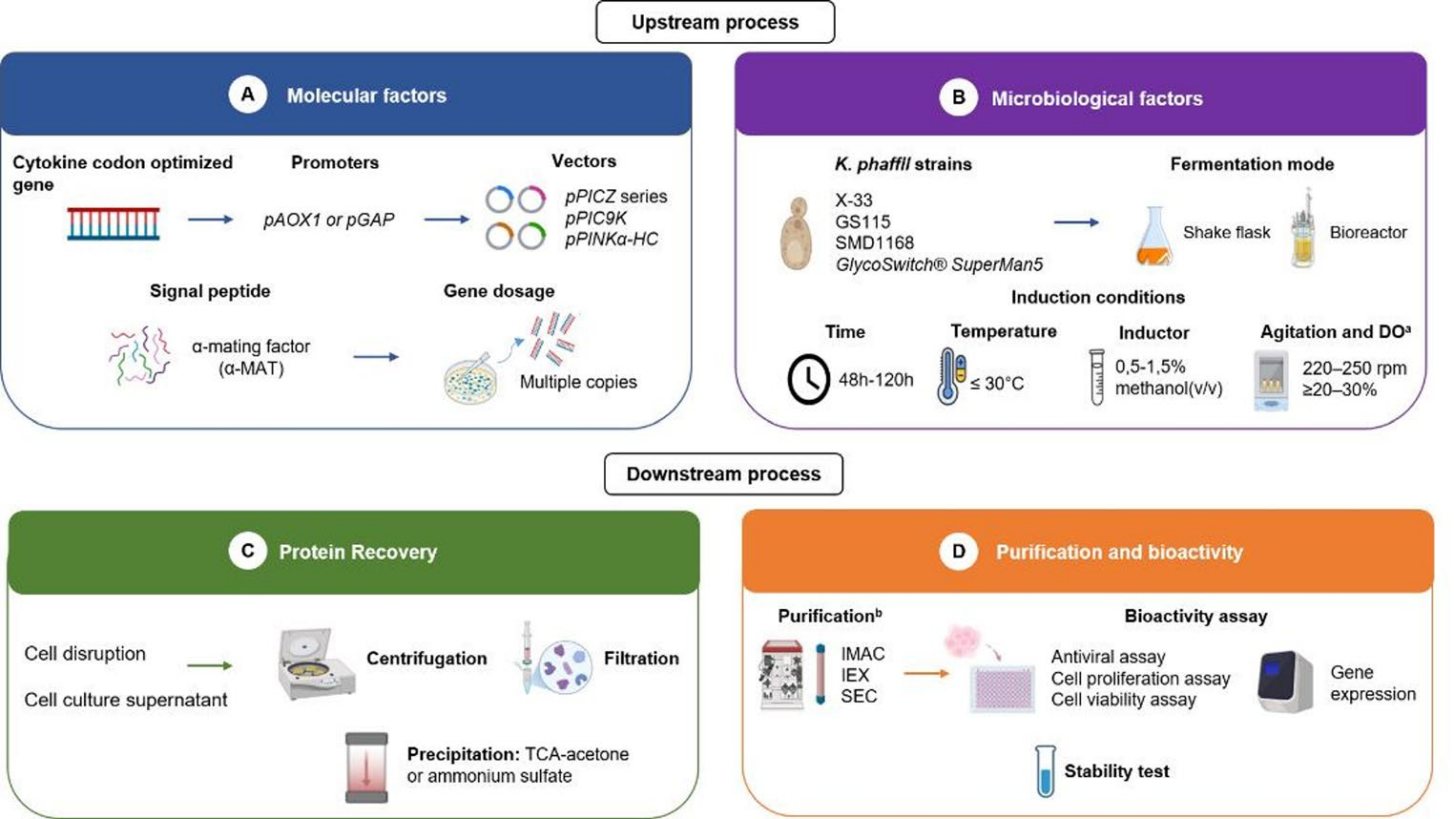
Summary of upstream and downstream processing for recombinant cytokine production in *K. phaffii*; ^a^DO: Dissolved oxygen maintained at 20–30% saturation; ^b^Chromatographic purification methods, including IMAC, immobilized metal affinity chromatography; IEX, ion-exchange chromatography; SEC, size-exclusion chromatography

Recombinant protein production in *K. phaffii* involves two main phases: upstream and downstream processing (Fig. 1). The upstream phase encompasses all stages related to molecular design, host cell cultivation, and process optimization to achieve efficient expression of the target protein. In yeast-based systems, productivity is influenced by several genetic and environmental factors, including codon usage optimization, gene copy number, promoter strength, secretion signals, host strain selection, and culture parameters such as temperature, pH, dissolved oxygen, and inducer concentration [10]. The following subsections summarize and discuss the main molecular and bioprocessing strategies used to enhance cytokine expression in *K. phaffii*.

### Expression vectors and promoters

Efficient expression of recombinant proteins relies on vectors that integrate specific regulatory elements to ensure high-level transcription and translation of the target gene. Several expression vectors have been developed for use in *K. phaffii*, differing mainly in their promoter type, selection marker, and compatibility with host strains.

The alcohol oxidase I (*AOX1*) promoter (*pAOX1*) is the most commonly used and is strongly induced by methanol, enabling tight regulation of expression [13]. Alternatively, constitutive promoters such as *pGAP*, derived from the glyceraldehyde-3-phosphate dehydrogenase gene, allow continuous expression without the need for inducers [14, 15]. However, *pGAP* often results in lower yields, limiting its industrial applicability [16].

Among the most widely used vectors for cytokine expression are the pPICZ series (pPICZαA, pPICZαB) and pPIC9K. Both are designed for secretion but differ in selection strategy and application. The *pPIC9K* vector includes dual resistance genes—*kanR* and *G418*—allowing selection of multicopy clones with elevated expression levels, though it requires auxotrophic strains such as GS115 and selection in histidine-deficient media [16]. In contrast, *pPICZ* vectors carry a zeocin resistance marker, are smaller and easier to manipulate, and support multiple host strains [16]. Other systems, such as PichiaPink™, employ *pPINKα-HC* vectors and facilitate visual identification of multicopy clones via ADE2 knockout, producing white (high copy) and pink (low copy) colonies [17, 18].

### Secretion signals and protein export

One major advantage of *K. phaffii* is its ability to secrete recombinant proteins into the culture medium, which simplifies purification since the yeast produces relatively few endogenous secreted proteins [19]. Efficient secretion depends on a leader sequence that directs the nascent peptide to the secretory pathway.

The α-mating factor (α-MAT) signal from *Saccharomyces cerevisiae* is the most widely used secretion signal and is already incorporated into commercial *K. phaffii* vectors [20]. However, secretion efficiency varies depending on the target protein. To improve secretion of porcine IFN-α, He et al. [21] used a synthetic signal peptide (MF4I), a modified α-pre-leader sequence from *S. cerevisiae*, resulting in higher secretion yields. Similarly, Aggarwal and Mishra [22] reported that deletion of the pro-region in α-MAT increased cytokine expression twofold, and later demonstrated that mutations near the Kex2 cleavage site enhanced extracellular accumulation of G-CSF [23]. These findings underscore that modifying signal peptides can significantly improve secretion efficiency and product yield.

### Codon optimization and gene copy number

Codon optimization is one of the most effective molecular strategies for improving heterologous expression in *K. phaffii*. This approach involves replacing rare codons with synonymous ones preferred by the host, aligning GC content, and stabilizing mRNA secondary structure [5]. Optimized constructs typically lead to increased transcriptional efficiency and higher yields of functional protein [24–26]. For instance, optimized bovine IFN-α achieved higher expression levels than the native sequence at equivalent gene copy numbers [27], and similar improvements were reported for human IFN-γ [28] and feline IFN-ω [29].

Gene dosage also has a pronounced impact on expression. Liang et al. [30] demonstrated that recombinant strains carrying multiple tandem copies of the IFN-γ gene exhibited significantly higher expression levels. He et al. [21] similarly reported enhanced secretion of porcine IFN-α with six integrated gene copies. However, Dagar and Khasa [10] observed that excessive gene dosage reduced IL-3 expression, likely due to metabolic burden and competition for transcriptional and translational resources. Therefore, optimal gene copy number must be balanced to maximize expression without imposing cellular stress.

### Host strain selection and cultivation parameters

The choice of host strain and culture conditions is crucial for optimizing cytokine yields. Commonly used strains include X-33 (wild-type) and GS115, which is auxotrophic for histidine due to a *HIS4* mutation. Both grow rapidly on methanol and are widely used for cytokine expression [9]. However, proteolytic degradation of recombinant proteins remains a challenge [5]. Protease-deficient strains, such as SMD1168 (*Δpep4*,* Δprb1*), minimize degradation and have been successfully used for cytokine expression [31–33], though protease inhibitors are often still required, increasing production costs.

The PichiaPink™ system offers additional advantages, with strains carrying single (*Δpep4* or *Δprb1*) or double (*Δpep4/Δprb1*) deletions, enabling faster clone selection and higher copy number integration [17, 18, 34]. For therapeutic cytokines requiring human-like glycosylation, glycoengineered strains such as GlycoSwitch^®^ SuperMan5 have been developed. These strains perform Man5-type N-glycosylation and are also protease-deficient, enhancing both stability and bioactivity of proteins such as rhIFN-α2b [33, 35–37].

Culture conditions also play a vital role. Temperature, pH, methanol concentration (v/v), and dissolved oxygen (DO) levels strongly influence productivity [32]. Lower induction temperatures (22–26 °C) often improve folding and reduce proteolysis [18, 26–28, 38–41]. Optimal methanol concentrations typically range from 0.5% to 1.5% (v/v), with higher levels causing toxicity and lower levels reducing induction efficiency [10, 28, 38, 40, 42–44]. Co-feeding strategies using methanol with non-repressive substrates such as sorbitol have been particularly successful, improving energy metabolism and yield by 1.3-fold compared to methanol-only conditions [38, 45].

### Integrated overview

In *K. phaffii*, achieving high recombinant protein yields depends on a complex interplay of genetic and physiological parameters, including codon optimization, gene copy number, promoter strength, signal peptide efficiency, and the fine-tuning of cultivation variables such as temperature, pH, and dissolved oxygen concentration [10]. Each of these elements can markedly influence expression levels and secretion efficiency. A consolidated overview of the main upstream strategies reported for recombinant cytokine production in *K. phaffii* encompassing vector design, strain selection, and induction conditions is presented in Table 1.

### Downstream processing and purification strategies

After the expression of the target protein, downstream processing becomes a critical stage to recover and purify the recombinant cytokine with high yield and biological activity [46]. Because *K. phaffii* secretes few endogenous proteins, the supernatant contains relatively low background contamination, simplifying purification and reducing costs. Efficient downstream design is essential to ensure that each cytokine is recovered with adequate quality, purity, and scalability for potential therapeutic use [12, 47]. Figure 1 summarizes the principal purification strategies reported for cytokines expressed in *K. phaffii*.

### Recovery and primary purification

Initial recovery of proteins secreted into the culture medium typically involves centrifugation, ultrafiltration, or precipitation. Ammonium sulfate precipitation is widely employed as a salting-out method due to its high solubility and ability to concentrate proteins without denaturation. Optimal saturation levels vary across studies and must be empirically determined for each cytokine [24, 26, 30, 47–51]. For example, Sembiring et al. [51] reported that 80% saturation maximized recovery of rhGM-CSF, while Pykhtina et al. [26] used 75% saturation for rGM-CSF and its ApoA-I fusion. Liang et al. [30] found 60% saturation optimal for precipitating rIFN-γ fused to HBscFv, and Miroshnichenko et al. [50] used 40–50% saturation for rIFN-α2b and its ApoA-I co-expression variant. These studies highlight that higher ammonium sulfate saturation levels (75–80%) tend to enhance protein recovery, likely due to increased precipitation efficiency. However, this approach may also co-precipitate a greater amount of contaminant proteins, reducing purity and requiring additional downstream purification steps. In contrast, lower saturation levels (e.g., 50%) may improve selectivity, favoring the precipitation of the target protein while leaving more impurities in solution, but often at the cost of reduced overall yield. Therefore, the choice of saturation level represents a trade-off between recovery and purity, depending on the intended downstream application.

### Chromatographic purification

Cytokines, particularly those intended for therapeutic applications, must meet the stringent purity standards of the biopharmaceutical industry, often exceeding 99% while retaining biological functionality [12]. Several chromatographic techniques have been employed depending on the physicochemical properties of each protein, such as size, charge, and hydrophobicity.

### Affinity chromatography

Immobilized metal affinity chromatography (IMAC) using Ni–NTA is the most common approach for histidine-tagged cytokines. Prabhu et al. [52] purified extracellular rhIFN-γ from bioreactor supernatant, obtaining 83% purity with 53% recovery. Zhang et al. [39] achieved 95% purity for mink rIFN-α using a ten-fold concentrated supernatant. Although IMAC is efficient, additional steps are often required for higher purity. For example, Katla et al. [33] purified N-glycosylated hIFN-α2b via Ni–NTA followed by lectin-based affinity chromatography using Concanavalin A (ConA), isolating the glycosylated fraction at 95% purity. Alternative affinity ligands have also proven effective: Naseem et al. [53] employed a human serum albumin (HSA)-binding column to purify an rcIFN–HSA fusion (98% purity), and Ningrum et al. [31] used Blue Sepharose 6 Fast Flow with cibacron blue to purify both native and HSA-fused rhIFN-α2a (74% recovery). Liang et al. [30] achieved 95–98% purity of HBscFv–IFN-γ through immunoaffinity chromatography with a monoclonal antibody against HBscFv.

### Ion-exchange chromatography

Ion-exchange chromatography (IEX) separates proteins based on electrostatic interactions between charged protein residues and oppositely charged resins [54]. Depending on the buffer pH relative to the protein’s isoelectric point, either cationic or anionic exchange can be employed. Pykhtina et al. [24] purified human rhG-CSF in a two-step process using DEAE- and SP-Sepharose FF, reaching 95% purity but below 50% yield. The same group applied this strategy to rhGM-CSF and its ApoA-I fusion with similar purity [26]. Miroshnichenko et al. [50] obtained 95–98% purity and 68% recovery for rIFN-α2b using DEAE- and SP-Sepharose resins, a purity level comparable to that reported by Katla et al. [33], who employed affinity chromatography to purify N-glycosylated rIFN-α2b. While ion-exchange chromatography (IEX) offers high capacity and cost-effectiveness, it generally requires multiple steps to achieve high purity; in contrast, affinity chromatography provides greater selectivity and can simplify the purification workflow, often leading to improved recovery, although at a higher cost and potential need for protein modification. Similarly, An et al. [42] reported 85% recovery and 92% purity for rIFNλ-3 using Q-Sepharose FF, while Barathiraja et al. [55] purified the same cytokine with SP-Sepharose, yielding 23.9 mg/L. These studies highlight IEX as a powerful complementary step to affinity chromatography, enabling fine charge-based separation and serving as an effective polishing strategy to enhance purity after initial capture.

### Size-exclusion chromatography

Size-exclusion chromatography (SEC), or gel-filtration, is frequently employed as a final polishing step to remove aggregates and residual contaminants while preserving protein conformation [11]. Jia et al. [56] used SEC with a HiPrep Sephacryl S-200 resin after MabSelect affinity chromatography to purify rIFN-α/Fc fusion proteins, achieving > 90% purity. Ningrum et al. [43] compared SEC and affinity methods for rhIFN-α2a and its HSA fusion; although SEC yielded low impurities, the affinity method provided better recovery and was selected as the preferred strategy. This result reinforces that, although SEC is effective in reducing impurities and improving sample homogeneity, its low selectivity and dilution effects may limit overall recovery, whereas affinity chromatography enables selective capture and concentration of the target protein, resulting in higher yields and a more efficient downstream process.

### Alternative extraction techniques

Beyond conventional chromatography, novel extraction systems have been explored to simplify downstream operations. Prabhu et al. [47] developed a nickel-chelated reverse micellar extraction (RME) technique for rhIFN-γ, achieving 67% recovery and 79.5% purity under optimized conditions determined through statistical modeling. RME combines the selectivity of affinity interactions with the scalability of liquid–liquid extraction, offering a promising alternative for industrial processes; however, these results differ from those obtained by Prabhu et al. [52] using IMAC, which achieved higher purity but lower recovery, highlighting that while IMAC provides greater selectivity and more consistent purity, RME may offer improved recovery and process integration, albeit with reduced purification efficiency and the need for additional polishing steps.

### Bioactivity validation and product characterization

The biological activity of recombinant cytokines depends on purity, correct folding, and structural integrity. Therefore, functional assays are essential after purification to confirm the preservation of biological activity. Typical evaluations involve activation of specific cell signaling pathways or quantification of defined cellular responses. Barathiraja et al. [55] showed that purified rIFNλ-3 induced expression of interferon-stimulated genes (ISGs) in bovine PBMCs, confirming its bioactivity. Prabhu et al. [47] demonstrated that rhIFN-γ obtained through RME reduced MCF-7 cell viability by approximately 25% at higher concentrations, and maintained antitumor activity in oral cancer cell lines. Dagar and Khasa [10] confirmed that rhIL-13 remained active in TF-1 cells by MTT assay, while Shao et al. [57] and Wang et al. [29] verified strong antiviral activity of rIFN-α14 and feline rIFN-ω, respectively. Together, these results confirm that proteins produced in *K. phaffii* retain their expected biological function after purification, validating the system’s potential for producing cytokines suitable for biomedical and veterinary applications.

### Process integration and optimization

To obtain a final product of adequate quality and yield, it is essential to employ downstream strategies that are efficient, scalable, and economically viable. The selection of precipitation, affinity, ion-exchange, or size-exclusion chromatography techniques depends on the physicochemical properties of each cytokine and its intended application. A summary of the main downstream recovery reported for recombinant cytokines expressed in *K. phaffii* is provided in Table 1.

**Table 1:** Upstream and dowstream processing parameters and molecular strategies used for recombinant cytokine production in *Komagataella phaffii*

Cytokine	Upstream process						Dowstream process		Reference
	Strain/plasmid	Promoter		Codons optimization	Signal peptide	Induction parameters (time, temperature, % inductor v/v)	Concentration	Biological Activity	
hG-CSF	X-33/pPICZαB	AOX1		Yes	α-factor modified	28 °C; 120 h; 1% methanol	60–100 mg/L^**a**^	NA	[22]
hG-CSF	X-33/pPICZαB	AOX1		Yes	α-factor modified cleavage site	28 °C; 120 h; 1% methanol	22 mg/L (wild peptide) and 60–100 mg/L (mutaded) ^**a**^	NA	[23]
hG-CSF	X-33/pPICZαB	AOX1		NA	α-factor	28° and 22 °C; 96 h; 1% methanol	1.33 mg/L^**a**^	Cell Proliferation Assay: Biologically Active	[70]
hG-CSF	X-33/pPICZαB	AOX1		Yes	α-factor	Fed-batch fermentation with glycerol, methanol, and sorbitol; 96 h	NA	NA	[71]
hG-CSF	GS115/pPICZα	AOX1		NA	α-factor	30 °C; 48 h; 0,5% methanol	29.6 mg/L^b^	NA	[51]
hG-CSF	X-33/pPICZαA	AOX1		Yes	α-factor	26 °C; 72 h; tween 20 and 1% methanol	35 mg/L^b^	BMCS Cell Analysis: Biologically active	[24]
G-CSF and IFN-α	G115 and KM71/NA	NA		NA	NA	Fermentation with different induction strategies high DO/low MeOH and high MeOH/low DO with methanol and sorbitol; 70 h	1.86 mg/L (high DO/low MeOH strategy) ^**a**^	NA	[45]
hGM-CSF	X-33/pPICZαA	AOX1		Yes	α-factor	25 °C; 96 h; 0,2% tween 20 and 1% methanol	100 mg/L (ryGM-CSF) e 60 mg/L (ryGM-CSF-ApoA-I)^b^	Cell Proliferation Assay with TF-1 cells and BMCs: Biologically active	[26]
hGM-CSF	Pichiapink/pEP(α)101	FMD		Yes	α-factor	30 °C; 48 h	364 mg/L; 323 mg/L and 271 mg/L^**a**^	NA	[34]
mGM-CSF	KM71H/pPICZαA	AOX1		Yes	α-factor	25 °C; 120 h; with addition of methanol (does not mention concentration)	18.5 mg/mL^**a**^	Murine Factor-Dependent FDC-P1 Growth Factor Stimulation Assay: Biologically Active	[72]
hIFN	X-33/pPICZαA	AOX1		Yes	α-factor	25 °C; 96 h; 0,2% tween 20 and 1% methanol	NA	Antiviral Assay Against *equine vesicular stomatitis virus* and SARS-CoV-2: Biologically Active	[50]
hIFN-γ	GS115/pPICZα	AOX1		NA	α-factor	25 °C; fed batch with addition of methanol and glucanol	NA	NA	[69]
fIFN-ω	GS115/pPICZαA	AOX1		Yes	α-factor	120 h; 0,5% methanol	NA	Antiviral Assay Against *VSV* and *FPV* (In vitro) and In vivo Assay with Infected Animals: Biologically Active	[29]
cIFNα	GS115/pPICZαA	AOX1		NA	α-factor	96 h; 0,5% methanol	23 mg/L^b^	Antiviral Assay Against *VSV* and *Canine Influenza Virus*: Biologically Active	[60]
bIFNα	GS115/pPIC9K	AOX1		Yes	α-factor	20, 24, 26, 28, 30 °C; 72 h; 1% methanol	200 mg/L^b^	Antiviral Assay in MDBK and IBRS-2 Cells: Biologically Active	[27]
hIFN-α	GS115/pPICZαB	AOX1		Yes	α-factor	30 °C; 96 horas; 0,5% methanol	NA	Cytopathic Inhibition Assay: Biologically Active	[53]
hIFN-α	GS115/pPIC9K	AOX1		NA	α-factor	25 °C; fed batch with methanol	NA	Antiviral Assay with WISH or MDBK Cells and Anti-cell Proliferation Assay: Biologically active	[56]
vIFN-α	X-33/pPICZαA	AOX1		Yes	α-factor	30 °C; 72 h; 0,8% methanol	95 mg/L^b^	Antiviral Assay with F81, Wish, and Vero Cells Against *VSV*: Biologically Active	[39]
pIFN-α	GS115/pBDM	AOX1		Yes	MF4I	28 °C; 84 h; 1% methanol	17,000 mg/L^**a**^	Antiviral Assay with MDBK Cells against *VSV*: Biologically active	[21]
pIFN-α	KM71/pPICZα	AOX1		NA	α-factor	30 °C and 20 °C; fed batch with methanol and sorbitol	2,700 g/L (methanol/sorbitol strategy 20 °C); 2,100 g/L (methanol and sorbitol 30 °C) and 1,100 g/L (methanol 20 °C) ^**a**^	Antiviral Assay: Biologically Active	[38]
bIFN-α1	GS115/pPICZαA	AOX1		NA	α-factor	28 °C; 72 h; 0,5% methanol	0.2 mg/L^**a**^	Antiviral Assay with MDBK Cells Against *VSV*: Biologically Active	[57]
bIFN-α₁₄	GS115/pPICZαA	AOX1		NA	α-factor and cytokine signal peptide	Does not specify temperature or methanol concentration/ 96 h	NA	Antiviral Assay with MDBK Cells Against *VSV* and Antiproliferative Assay in MDBK cells: Biologically Active	[61]
IFNα-16 and IFN-γ	GS115 and GS115-modificaded/ pPICZαA	AOX1		NA	α-factor	Two-stage strategies: 30 °C and 20 °C (2 stages) and 25 °C (1 stage); 48 h; do not mention methanol induction	1st stage: 1.2 mg/L (hIFNα16) and 0.7 mg/L (chkIFN-γ); 2st stage: 1.3 mg/L (hIFNα16) and 1.5 mg/L (chkIFN-γ)^**a**^	NA	[73]
hIFNα2a	Pichiapink/pPinkα-HC	AOX1		Yes	α-factor	22 °C; 72 h; 1% methanol	NA	Evaluation of the Induction Potential of Interferon-Stimulated Genes (ISGs) in A549 and HT29 cells	[18]
hIFNα2a	GS115 and SMD1168/NA	AOX1		NA	NA	30 °C; 48 h; 1% methanol	14 mg/L (fused protein) and 44 mg/L (no fusion)^b^	NA	[31]
hIFNα2a	GS115 and SMD1168/pPICZαB		AOX1	Yes	α-factor	30 °C; 24 and 48 h; 0,5% methanol	14 mg/L^b^	Antiproliferative Assay with MCF-7 cells: Reduced biological activity compared with non-fusion protein	[32]
hIFNα2a	GS115/pPICZαB	AOX1		NA	α-factor	28 °C; 48 h; 0,5% methanol	44 mg/L^b^	NA	[64]
hIFNα2a	GS115/pPICZαB	AOX1		NA	α-factor	30 °C; 48 h; 1,5% methanol	0,272 mg/L^b^	Antiproliferative Assay with MCF-7 Cells: Biologically Active	[65]
hIFNα2a	GS115 and SMD1168/NA	NA		NA	NA	27 and 30 °C; 24, 48, 72 h, 0.5–2.5% methanol	16 mg/L (fused protein) and 48 mg/L (no fusion)^b^	NA	[43]
hIFNα2b	SuperMan5/NA	AOX1		NA	α-factor	Fed batch with glycerol, methanol and ammonium sulfate	436 mg/L^b^	Antiproliferative Assay with MCF7 Cells: Biologically Active	[37]
hIFNα2b	SuperMan5/NA	AOX1		NA	α-factor	Different strategies for fed-batch	NA	NA	[67]
hIFNα2b	SuperMan5/NA	NA		NA	NA	Fed batch with glycerol, methanol and sorbitol	288 mg/L^**a**^	NA	[36]
hIFNα2b	SuperMan5/pPICZα	AOX1		NA	α-factor	30 °C; Fed batch with methanol	NA	NA	[74]
hIFNα2b	X-33/NA	AOX1		NA	NA	30 °C; 48 h; 2% methanol	10.92 mg/L^b^	Antiproliferative Assay with MCF7 Cells: Reduction in Activity	[66]
hIFNα2b	X-33/NA	NA		NA	NA	30 °C; 0,5% methanol	NA	Antiproliferative Assay with MCF7 Cells: Biologically Active	[44]
hIFNα2b	SuperMan5/NA	AOX1		NA	α-factor	Different bioprocess strategies and simple and robust feedback control based on PAT	NA	Antiproliferative Assay with T47D and MCF7 Cells: Biologically Active	[35]
hIFNα2b	X33 and SuperMan5/pPICZαA	AOX1		Yes	α-factor	30 °C; 72 (bioreactor) and 96 h (small scale); 0,5% methanol	350 mg/L (SuperMan5) and 320 mg/L (X33)^b^	Antiviral Assay using the Subgenomic Replicon Assay Systems for *HCV* and *HEV*: Biologically Active	[33]
hIFNα2b	GS115/pPICZαA	AOX1		NA	α-factor	28 °C; 120 h; 1% methanol	15–17 mg/L^b^	Cytopathic Effect Inhibition Assay Against *EMC* virus in Human Lung Carcinoma Cell Line and Antiviral Activity Assay Against *HCV genotype 3a*: Biologically Active	[48]
hIFN-β-1a	GS115/pPICZαA	AOX1		NA	α-factor	28 °C; 72 h; 2% methanol	NA	NA	[41]
hIFN-γ	GS115/pPICZB and pKANB	AOX1		Yes	α-factor	30 °C; Fed batch with methanol and glucanol	80 (IFN) and 123 mg/L (co-expressing other proteins)^**a**^	NA	[68]
pIFN-γ	X-33/pPICZαA	AOX1		Yes	α-factor	25 °C and 28 °C; 48–72 h; 0,5% methanol	536,4 mg/L^b^	Analysis of the Expression of *OAS1*, *Mx1* and *PKR* genes in IPEC-J2 Cells Cultured with the Cytokine	[75]
hIFN-γ	X-33/pPICZαB	AOX1		Yes	α-factor	25 °C; 1% methanol	36 mg/L^c^	Cell Viability Assay with MCF-7 Cells: Biologically Active	[47]
hIFN-γ	GS115/ pPICZαA and pPIC9K	AOX1		Yes	α-factor	20 °C, 25 °C, 28 °C and 37 °C; 72 h; 0,25 − 2% methanol	2.5mg/L^b^	NA	[28]
hIFN-γ	GS115/pPIC9K	AOX1		Yes	α-factor	28 °C; 72 h; 1% methanol	0.28 mg/L^**a**^	NA	[76]
hIFN-γ	GS115/pPIC9K and pPICZαB	AOX1		NA	α-factor	28 °C; 1% methanol	1.98 mg/L^b^	Cell Viability Assay with Oral Cancer Cells: Biologically Active	[52]
hIFN-γ	GS115/NA	NA		NA	NA	28 °C; Fed batch with methanol and glucose	NA	Cytotoxicity Assays	[77]
IFN-γ	X-33/pPICZαA	AOX1		NA	α-factor	28 °C; 72 h; 1% methanol	15 mg/L^b^	Neutralization Assay with ELISA and in *HBV* Transgenic Mice	[30]
fIFN-γ	NA/pPICZαA	AOX1		Yes	α-factor	28 °C; 96 h; 1% methanol	242 mg/L^**a**^	WISH Cell Assay: Biologically Active	[49]
bIFN-λ3	GS115/pPICZαA	AOX1		Yes	α-factor	28 °C; 96 h; 0,5 − 4% methanol	Precipitation: 152,1 mg/L (boIFN–λ3) and 148,5 mg/L (boIFN–λ3V18M)^**a**^; Ion exchange: 29,440 mg/L (boIFN–λ3) and 31,450 mg/L (boIFN–λ3V18M)^b^	Antiviral and Antiproliferative Assay with MDBK cells: Biologically Active	[42]
bIFN-λ3	GS115/pPICZαA	AOX1		NA	α-factor	96 h; 1% methanol	23.92 mg/L^b^	Assay with bovine PBMCs: Biologically Active	[55]
bIFN-ω	GS115/pPICZαA	AOX1		Yes	α-factor	30 °C; 96 h; 1% methanol	1,542 mg/mL^**a**^	Antiviral and Antiproliferative Assay with MDBK cells: Biologically Active	[62]
hIL-11	PichiaPink/pPINKα-HC	AOX1		Yes	α-factor	30 °C; 72 h; Fed batch with methanol	NA	Cell Proliferation Assay in 7TD1 cells	[17]
hIL-15	X-33/pPICZαA	AOX1		NA	NA	28 °C; 96 (large scale) and 120 h (small scale); 1% methanol	75 mg/L^b^	CTLL-2 and NK Cell Proliferation Assay	[78]
hIL-15	GS115/pPICZα and pPIC9K	AOX1		NA	α-factor	25 °C; 43–45 h; Fed batch with methanol	NA	Cell Proliferation Assays and In vivo Assays: Biologically Active	[59]
bIL-15	GS115/pPICZαA	AOX1		NA	α-factor	30 °C; 96 h; 0,5% methanol	NA	Bovine PBMC Cell Assay to measure immune-related genes: Biologically Active	[58]
pIL-18	NApPS10	AOX1		NA	NA	30 °C; 72 h; 1% methanol	NA	Lymphocyte Cell Proliferation Assay and Immunostimulation in Pigs: Biologically Active	[79]
IL-18	KM71/pPICZαA	AOX1		NA	α-factor	30 °C; 48 h; 2% methanol	NA	Interferon-γ Induction Assay: Biologically Active	[80]
hIL-1B	X-33, GS115 and SMD1168/pPICZαA	AOX1		Yes	α-factor	20 °C, 23 °C, 26 °C and 29 °C; 72 h; 0,25 − 1% methanol	NA	Antiproliferative Assay with B16 Melanoma Cells: Biologically Active	[40]
pIL-2	SMD1168/pGAPZαA	GAP		NA	α-factor	30 °C; 72 h	NA	Antimicrobial Assay and Swine Lymphocyte Proliferation Assay	[15]
IL-2/4 and 6	SMD1168/pGAPZαA	GAP		NA	α-factor	30 °C	NA	In vivo Assay with pigs and Analysis of Immune response	[14]
hIL-22	GS115/pPIC9k	AOX1		Yes	α-factor	30 °C; 72 h; 0,5% methanol and ascorbic acid and NAC	2,250 mg/L^c^	Proliferative Assay with HepG2 cells: Biologically Active	[81]
fIL-26	X-33/pPICZαA	AOX1		NA	NA	29 °C; 48 h	NA	Assays to Evaluate Cytokine Expression: Conserved Activity	[82]
hIL-29	GS115/pPIC9k	AOX1		NA	α-factor	30 °C; 72 h; 2% methanol	3.6 mg/L (K-33R), 3.2 mg/L (R35K) and 4.4 mg/L (K-33R/R35K)^b^	Evaluation of Antitumor Activity	[83]
hIL-3	GS115/pPICZαA	AOX1		Yes	α-factor	96 h; 0,5% methanol	135 mg/L (Native), 64.36 mg/L (N15A), 85.74 mg/L (N70A) and 38.59 mg/L (N15/70A)^b^	Antiproliferative Assay with TF-1 cells: Biologically Active	[84]
hIL-3	X-33/pPICZαA	AOX1		Yes	α-factor	Small scale: 30 °C; 84 h; 0,5–5% methanol; Large scale: Fed-batch with methanol	350 mg/L (established optimal parameters); 2.23 g/L (batch fed with basal saline medium)^b^	Antiproliferative Assay with TF-1 cells: Biologically Active	[10]
fTNF-α	SMD1168/pPICZαA	AOX1		NA	α-factor	1% methanol	NA	Immune Gene Expression Assays	[85]

^a^Protein concentration obtained after processing by centrifugation, ultrafiltration, or precipitation with acetone or ammonium sulfate;

^b^Protein concentration after an additional purification step by chromatography;

^c^Protein concentration after an additional purification using a non-chromatographic method

### Therapeutic and experimental applications of recombinant cytokines

Cytokines produced in *K. phaffii* have been extensively evaluated for biomedical and veterinary applications due to their immunomodulatory, antiviral, and hematopoietic properties. The yeast-based expression system allows the generation of biologically active cytokines with proper folding and post-translational modifications, while offering scalable and cost-efficient production. The following subsections summarize major advances in the expression and characterization of interleukins, interferons, and colony-stimulating factors, as well as molecular strategies developed to enhance their stability and therapeutic performance.

### Interleukins

Interleukins (ILs) are key regulators of immune signaling and hematopoiesis. Several ILs were successfully expressed in *K. phaffii*, including IL-2, IL-3, IL-4, IL-11, IL-13, IL-15, and IL-18. These molecules have been used to modulate immune responses, support T-cell proliferation, and serve as adjuvants in vaccine development.

Among them, IL-15 stands out for its ability to promote the survival and expansion of NK and CD8⁺ T cells. Different expression strategies have been explored to improve its biological performance. Vijay et al. [58] expressed mature human IL-15 in *K. phaffii* and demonstrated higher bioactivity than the bacterially produced form, as shown by increased Bcl-2 expression, STAT3 phosphorylation, and activation of memory CD8⁺ T cells. This difference likely reflects the advantages of the yeast expression system, which can provide eukaryotic folding and post-translational processing that are absent in bacterial hosts. In contrast, Xu et al. [59] focused on improving IL-15 pharmacokinetics rather than intrinsic activity by engineering fusion constructs containing the soluble IL-15 receptor α (SuIL-15Rα) and the Fc region of IgG4. These constructs retained full biological activity while significantly prolonging serum half-life. Together, these studies illustrate how distinct strategies such optimization of the expression host versus protein fusion design can enhance different functional aspects of IL-15, including bioactivity and in vivo stability.

Other interleukins also showed functional activity when expressed in *K. phaffii*. Dagar and Khasa [10] produced human IL-13 in a multicopy strain and confirmed its biological activity in TF-1 cells via MTT assay. Ningrum et al. [34] expressed human IL-11, which was properly folded and secreted into the medium. These findings underscore the yeast’s capacity to produce functional, structurally complex cytokines suitable for immunotherapeutic applications.

### Interferons

Interferons (IFNs) constitute the first line of defense against viral infections and play key immunomodulatory roles [11]. The development of genetically modified IFNs with enhanced activity and reduced cost is of major interest for human and veterinary medicine. Recombinant interferons expressed in *K. phaffii* have been characterized in multiple species, including humans, cattle, pigs, dogs, cats, and minks [29, 38, 39, 60].

In cattle, IFN-α1, IFN-α14, IFN-λ3, and IFN-ω have demonstrated potent antiviral activities and therapeutic potential [27, 42, 57, 61, 62]. Porcine rIFN-α has also been optimized for use as a vaccine adjuvant [21]. In humans, interferons are employed for treating inflammatory diseases, hepatitis, and certain cancers [63]. Recombinant IFNα-2a and IFNα-2b are among the most widely used, and various strategies have been explored to improve their production and stability, such as expression without affinity tags [64, 65] or fusion with human serum albumin (HSA) [31, 32, 43].

The functional stability of rIFNα-2b was investigated by Wathon et al. [66], who demonstrated that parameters such as temperature significantly influence the recombinant protein’s activity. To enhance protein stability and activity, Katla et al. [33] expressed a glycoengineered IFNα-2b with a human-like N-glycosylation profile, providing the dual advantage of preserving full antiviral activity while improving pharmacokinetic properties, particularly by extending plasma half-life. However, this approach requires more advanced strain engineering. To optimize the production of such glycoengineered proteins, bioprocess engineering has further optimized production through real-time monitoring, sensor integration, and the use of artificial intelligence to model metabolic dynamics [35, 36, 67]. Coexpression of molecular chaperones such as PDI, KAR2p, SSA1p, and YDJ1p has been shown to increase interferon yields [28, 47], while modulation of the pentose phosphate pathway and fine-tuning of fermentation parameters enhance productivity [68, 69]. These studies demonstrate that no single strategy is universally optimal, and the choice depends on balancing yield, structural integrity, biological activity, and scalability.

### Colony-stimulating factors (CSFs)

Colony-stimulating factors (CSFs) stimulate the proliferation and differentiation of hematopoietic progenitor cells and have broad therapeutic use in treating neutropenia and promoting bone marrow recovery [45, 70]. Recombinant human granulocyte-macrophage CSF (GM-CSF) and granulocyte CSF (G-CSF) have been successfully produced in *K. phaffii* and exhibit high biological activity. Pykhtina et al. [24, 26] expressed both native and ApoA-I-fused rhGM-CSF, achieving 95% purity and demonstrating strong immunostimulatory potential.

### Engineering strategies to improve cytokine pharmacokinetics

Despite maintaining functional activity, many recombinant cytokines exhibit limited stability and short serum half-lives, which restrict clinical applicability. To address this, various protein engineering and chemical modification strategies have been implemented. PEGylation—the covalent attachment of polyethylene glycol chains—reduces immunogenicity and extends circulation time. Similarly, albumin and antibody fusion technologies improve molecular stability by leveraging long-lived carrier proteins. Naseem et al. [53] generated a cIFN–HSA fusion with greater thermal stability and prolonged serum persistence compared to native cIFN, while Jia et al. [56] designed IFN-α/Fc fusion variants with comparable bioactivity to PEGylated IFN-α but superior pharmacokinetics. Liang et al. [30] produced a recombinant rIFN-γ fused to HBscFv, achieving improved stability and yield through multimeric gene integration.

Sigar et al. [70] engineered a stable G-CSF–HSA fusion that retained bioactivity and exhibited increased thermal stability, while Katla et al. [33] reported that glycoengineered rIFN-α2b displayed a 1.3-fold longer half-life than the *E. coli*-derived form, maintaining antiviral potency. These strategies illustrate how molecular fusion and glycoengineering can expand the therapeutic utility of yeast-expressed cytokines. While HSA and Fc fusions primarily enhance half-life and in vivo stability, and glycoengineering improves pharmacokinetics without major structural modifications, HBscFv fusion can also increase production yield; however, these strategies may increase molecular complexity and pose additional downstream processing challenges. Consequently, although these modifications expand the therapeutic utility of yeast-expressed cytokines, they often require more robust and tailored purification strategies to ensure product quality and functionality.

Such approaches demonstrate that integrating molecular design and bioprocess optimization in *K. phaffii* can yield recombinant cytokines that combine scalability, structural fidelity, and improved pharmacological performance—positioning this yeast as a viable alternative platform for next-generation cytokine therapeutics.

## Conclusions and future perspectives

This review highlights the significant progress achieved in the design, production, and purification of recombinant cytokines using *K. phaffii* as an expression platform. The yeast’s ability to combine high productivity with eukaryotic post-translational modifications has made it one of the most promising hosts for cost-effective biopharmaceutical manufacturing. Through the optimization of molecular and bioprocess parameters, such as codon usage, gene dosage, promoter regulation, secretion signals, cultivation conditions, and purification strategies, *K. phaffii* has been successfully employed for the expression of various biologically active cytokines, including interleukins, interferons, and colony-stimulating factors.

Despite these advances, challenges remain in achieving consistent glycosylation patterns, maintaining protein stability, and ensuring efficient scale-up for clinical-grade production. Continued improvements in strain engineering and glycoengineering will be essential to overcome these limitations. The emergence of synthetic promoters, genome-editing tools such as CRISPR/Cas9, and advanced omics-based modeling are expanding the molecular toolbox available for fine-tuning protein expression and secretion pathways in *K. phaffii*.

At the process level, integrating real-time bioprocess monitoring, sensor-based control systems to enhance reproducibility and streamline scale-up for industrial applications. Moreover, the development of continuous fermentation and downstream purification systems could further reduce costs and environmental impact.

Finally, the combination of yeast-based production with advanced protein engineering strategies, such as PEGylation, albumin fusion, and antibody conjugation, offers new avenues for creating recombinant cytokines with extended serum half-life and improved therapeutic efficacy. As these technologies converge, *K. phaffii* is expected to play an increasingly central role in the next generation of biopharmaceutical production, bridging the gap between laboratory research and large-scale clinical application.

## Data Availability

No datasets were generated or analysed during the current study.
